# Acceptability and Feasibility of a Low-Cost Device for Gestational Age Assessment in a Low-Resource Setting: Qualitative Study

**DOI:** 10.2196/34823

**Published:** 2022-12-27

**Authors:** Angela Koech, Peris Muoga Musitia, Grace Mkanjala Mwashigadi, Mai-Lei Woo Kinshella, Marianne Vidler, Marleen Temmerman, Rachel Craik, Peter von Dadelszen, J Alison Noble, Aris T Papageorghiou, The PRECISE Network

**Affiliations:** 1 Centre of Excellence in Women & Child Health Aga Khan University Nairobi Kenya; 2 Department of Obstetrics and Gynaecology Aga Khan University Nairobi Kenya; 3 Health Services Unit Kenya Medical Research Institute Wellcome Trust Research Programme Nairobi Nairobi Kenya; 4 Department of Obstetrics and Gynaecology University of British Columbia Vancouver, BC Canada; 5 Nuffield Department of Women's and Reproductive Health University of Oxford Oxford United Kingdom; 6 Department of Women and Children's Health King's College London London United Kingdom; 7 Department of Engineering Science University of Oxford Oxford United Kingdom; 8 Oxford Maternal & Perinatal Health Institute University of Oxford Oxford United Kingdom; 9 See Authors’ Contributions

**Keywords:** gestational age, gestation, gynecology, gynecologist, prenatal, antenatal, maternal, fetus, fetal, ultrasound, imaging, pregnancy dating, handheld, portable, trust, artificial intelligence, sub-Saharan Africa, Africa, low cost, LMIC, low income, feasibility, acceptability, AI, pregnancy, pregnant, maternity, women's health, obstetrics, obstetrician, rural, remote, remote location, misconception, eHealth, digital health

## Abstract

**Background:**

Ultrasound for gestational age (GA) assessment is not routinely available in resource-constrained settings, particularly in rural and remote locations. The TraCer device combines a handheld wireless ultrasound probe and a tablet with artificial intelligence (AI)-enabled software that obtains GA from videos of the fetal head by automated measurements of the fetal transcerebellar diameter and head circumference.

**Objective:**

The aim of this study was to assess the perceptions of pregnant women, their families, and health care workers regarding the feasibility and acceptability of the TraCer device in an appropriate setting.

**Methods:**

A descriptive study using qualitative methods was conducted in two public health facilities in Kilifi county in coastal Kenya prior to introduction of the new technology. Study participants were shown a video role-play of the use of TraCer at a typical antenatal clinic visit. Data were collected through 6 focus group discussions (N=52) and 18 in-depth interviews.

**Results:**

Overall, TraCer was found to be highly acceptable to women, their families, and health care workers, and its implementation at health care facilities was considered to be feasible. Its introduction was predicted to reduce anxiety regarding fetal well-being, increase antenatal care attendance, increase confidence by women in their care providers, as well as save time and cost by reducing unnecessary referrals. TraCer was felt to increase the self-image of health care workers and reduce time spent providing antenatal care. Some participants expressed hesitancy toward the new technology, indicating the need to test its performance over time before full acceptance by some users. The preferred cadre of health care professionals to use the device were antenatal clinic nurses. Important implementation considerations included adequate staff training and the need to ensure sustainability and consistency of the service. Misconceptions were common, with a tendency to overestimate the diagnostic capability, and expectations that it would provide complete reassurance of fetal and maternal well-being and not primarily the GA.

**Conclusions:**

This study shows a positive attitude toward TraCer and highlights the potential role of this innovation that uses AI-enabled automation to assess GA. Clarity of messaging about the tool and its role in pregnancy is essential to address misconceptions and prevent misuse. Further research on clinical validation and related usability and safety evaluations are recommended.

## Introduction

Knowledge of gestational age (GA) informs decisions in maternal and neonatal care [1], such as the use of corticosteroids in suspected preterm labor [2] and timing of delivery in postterm pregnancy and other pregnancy complications [3]. Reliable estimation of GA improves care by guiding these decisions and reducing unnecessary interventions. It also enables more accurate categorization of low-birth-weight babies into preterm or small for gestational age [4], impacting care for these babies [5,6] and improving reporting of perinatal outcomes [1,7,8].

In most high-income country settings, accurate pregnancy dating is provided routinely through early pregnancy ultrasound [9]. However, routine pregnancy ultrasound is rarely available in low- and middle-income country (LMIC) settings. In addition, other methods of GA assessment are likely to be less reliable: recall of last menstrual period (LMP) is generally poor [10], there is frequent late initiation of antenatal care (ANC) [7,11], and there is a higher prevalence of fetal growth restriction [12].

Setting up sustainable routine pregnancy ultrasound services in resource-constrained LMIC settings is often difficult, particularly in rural and remote locations [13]. ANC is largely provided by nurses and nurse-midwives and understaffing is frequent [14]. Radiologists, sonographers, and obstetricians are limited, located primarily in urban areas, and burdened with managing pregnancy complications and clinical emergencies [13]. Skills for GA assessment by ultrasound often require lengthy training programs, regular quality control, and close supervision, all of which are difficult to achieve and sustain [15]. In addition, conventional ultrasound equipment is expensive and requires regular maintenance and appropriate infrastructure such as a reliable continuous power supply.

**Figure 1:**
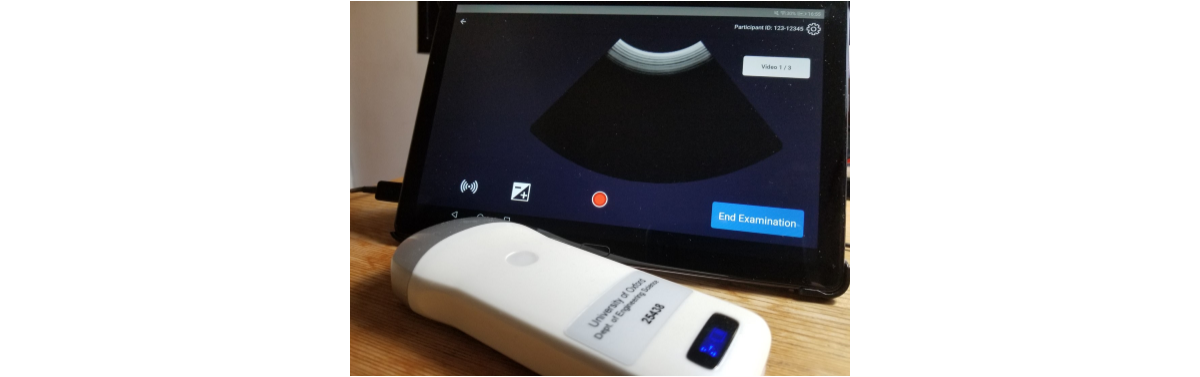
TraCer Device: the wireless handheld probe and TraCer software on the tablet.

The TraCer GA assessment device automates the measurement of the fetal transcerebellar diameter (TCD) and head circumference (HC). TraCer uses a low-cost, commercially available handheld battery-powered wireless ultrasound probe, which is linked, via Wi-Fi, to software running on a consumer-grade Android tablet (Figure 1). The device guides and assists health care workers (HCWs) to obtain ultrasound videos of the fetal head. GA is then estimated from the TCD and HC using semiautomated (and, in the future, automated) image recognition and analysis. The TCD estimates cerebellar size, which is considered a good measure for GA, as it is predictable throughout pregnancy and is not heavily impacted by the existence of fetal growth restriction [16,17]. The method also uses the fetal HC, because cerebellar imaging at advanced gestation stages may not always be possible using a low-cost device.

TraCer has been designed to specifically address challenges in resource-limited LMIC settings. However, this does not guarantee that it would be implementable or that communities and health care providers would find it acceptable [18]. A review of innovative approaches for improving maternal and newborn health found that gaps in understanding feasibility, appropriateness, and acceptability of implementation can compromise their capability for effective scale-up [19]. Therefore, it is recommended that new tools and innovations be evaluated not only for their technical and clinical performance, but also for acceptability and appropriateness, usability, and the feasibility of implementation within the intended settings [19].

Toward this end, in this study, we assessed the perceptions of pregnant women, their family members, HCWs, and managers regarding the acceptability and feasibility of the TraCer device in health facilities. This will guide further device development and inform plans for clinical implementation.

## Methods

### Study Design and Setting

A cross-sectional, descriptive, qualitative study was conducted in two public health facilities in Kilifi county in coastal Kenya. The two health facilities are the rural Rabai Health Centre (primary care facility) and the larger, urban Mariakani Sub-county Hospital (secondary care facility). Both facilities would later participate in the PRECISE (Pregnancy Care Integrating Translational Science, Everywhere) pregnancy cohort study [20]. At the time of data collection, enrollment to the PRECISE cohort had not yet started.

ANC, routine delivery care, and emergency care for pregnancy complications are provided primarily by nurses/nurse-midwives with support by clinical officers (nonphysician clinicians) at both facilities. At Mariakani Hospital, doctors and an obstetrician/gynecologist provide specialist services for high-risk pregnancies. Ultrasound services are not available routinely, but can be undertaken at Mariakani Hospital (and other private facilities) upon referral for pregnancy complications or uncertainties regarding GA.

### Study Participants and Sampling Methods

We sought to enroll two main groups of participants: (1) HCWs directly involved in the provision of services for pregnant women, as well as managers and health administrators; and (2) community members, represented by pregnant women participating in ANC and their family members (partners, as well as the pregnant woman’s parents and parents-in-law).

HCWs were purposely sampled to cover providers at the ANC clinic, maternity, outpatient, and radiology (including ultrasound) departments. Health administrators were also purposely sampled to ensure inclusion of facility and subcounty managers overseeing reproductive health services. Pregnant women were approached by research assistants when they presented for routine ANC, and participating women could invite their partners or parents.

### Data Collection

Data were collected between March and May 2019 by two Kenyan researchers: a social scientist (PMM) and a maternal health researcher and obstetrician (AK), who were assisted by two trained local research assistants who took notes during the sessions. Researchers were familiar with the local setting and the Kenyan health care system, and were fluent in both English and Swahili. The research assistants were also fluent in Mijikenda. None of the data collectors were involved in the participants’ clinical care; however, some HCWs had previous interactions with the two researchers as part of PRECISE study preparations.

In-depth interviews (IDIs) with HCWs and focus group discussions (FGDs) with pregnant women and their families were conducted in person in private areas of the health facility, away from the clinical areas. We developed a semistructured interview guide, which was piloted on two HCWs at Rabai Health Centre and revised prior to the subsequent interviews. The topic guide began with simple assessment of prior exposure to computers, smartphones, and obstetric ultrasound, followed by a discussion on existing methods of assessing GA and the potential value to pregnant women and HCWs.

The study was started before clinical implementation of TraCer. A video demonstrating its use during routine ANC was shown to participants. The 5-minute video, recorded at one of the facilities in Swahili, showed a nurse using TraCer with a pregnant woman who was unsure of her LMP. In the video, the nurse shows the mother the image of the fetal heartbeat and the head of the baby on the tablet screen, reports the GA, and then gives a date for the next clinic visit.

Participants were encouraged to voice their thoughts and ask any questions during and immediately after watching the video. Participants were asked what they liked or disliked about TraCer as seen in the video, whether TraCer could be introduced to their health facility, how confident they would be in its findings, any outcomes (positive or negative) they expected with its introduction, the type of provider they thought could use TraCer, and whether they would recommend it to other pregnant women and health facilities.

IDIs were conducted in the participants’ language of choice. HCWs preferred English, whereas pregnant women and their families preferred Swahili. All sessions were audio-recorded with permission and field notes were taken during each session. After each IDI and FGD, the research team debriefed to update field notes, discussed revisions and additional probes to the topic guide, and assessed data saturation.

### Data Analysis

All recordings were transcribed verbatim in the language of the interview and translated to English (where applicable) by research assistants. A sample of transcripts was compared with the recordings to ensure accuracy. NVivo 12 (QSR International, Melbourne, Australia) was used to manage the transcripts, and to facilitate coding and collaborative data analysis. The data analysis team comprised three Kenyan researchers familiar with the study site and local languages (AK, PMM, GMM), including two who had participated in the data collection (AK, PMM) and two experienced Canadian social scientists (MWK, MV). The data analysis team first familiarized themselves with the transcripts. Employing a directed content analysis approach [21], the coding framework was developed deductively from the research question based on pre-existing definitions of acceptability and feasibility [22] that were modified to fit our study. *Acceptability* was assessed according to the perceptions of the appropriateness of TraCer to participant needs, preferences, and sociocultural norms, along with factors that would influence willingness to use the device. *Feasibility* was assessed according to perceptions on whether TraCer could be implemented in the study health facilities and factors required for its successful implementation.

Major themes and subthemes were explored related to acceptability and feasibility. The coding framework (see Multimedia Appendix 1) was tested on three transcripts to refine and ensure agreement between coders. Transcripts were then divided between coders for analysis. Emergent common and divergent patterns of responses between participants were explored through discussion within the team. Factors that were considered included differences in the site characteristics (urban vs rural, level of facility, access to ultrasound), HCW characteristics (skill level/cadre and prior experience of ultrasound), and community member characteristics (age, gender).

### Ethical Considerations

The study obtained ethical approval from Aga Khan University Institutional Ethical Research Committee (2018_REC_47), King’s College London (Ref HR-17/18-7855), and University of British Columbia (H18-02828). All participants provided individual written informed consent prior to research activities. Confidentiality and safe storage of the data were ensured through deidentification of transcripts and electronic storage in password-protected devices accessible only to members of the research team.

## Results

### Characteristics of the Study Sample

In total, we conducted 18 IDIs and 6 FGDs involving 52 community members; the IDIs lasted 27-64 minutes and the FGDs lasted 34-77 minutes. In the IDIs, nurses represented the largest group interviewed, and other cadres were clinical officers, doctors, sonographers, and public health officials (Table 1). Seven of these HCWs also had administrative responsibilities. The 52 members engaged in the FGDs included 31 in three FGDs at Rabai Health Centre and 21 in three FGDs at Mariakani Hospital. Overall, FGDs engaged 19 pregnant women, 15 partners (all male), and 18 parents (all mothers or mothers-in-law). Fewer than 20% (9/52) of pregnant women or their families had prior experience of ultrasound (Table 2).

**Table 1:** Characteristics of health care workers participating in interviews (N=18).

Characteristics		Health care workers, n
**Site**		
	Rabai (rural)	8
	Mariakani (urban)	10
**Gender**		
	Male	7
	Female	11
**Profession**		
	Nurses	8
	Clinical officers	4
	Doctors	3
	Others	3
**Administrative responsibilities**		
	Yes	7
	No	11
**Age group (years)**		
	<35	7
	35-44	6
	45+	5
**Prior exposure to ultrasound (observing)**		
	Yes	14
	No	4
**Prior exposure to ultrasound (performing)**		
	Yes	3
	No	15

**Table 2:** Characteristics of pregnant women and their families participating in focus group discussions (N=52).

Characteristics		Pregnant women and their families, n
**Site**		
	Rabai (rural)	31
	Mariakani (urban)	21
**Gender**		
	Men	15
	Women	37
**Category**		
	Pregnant women	19
	Partners	15
	Parents and parents-in-law	18
**Age group (years)**		
	<35	30
	35-44	8
	45+	14
**Prior exposure to ultrasound^a^**		
	Yes	9
	No	43

^a^Refers to any exposure to ultrasound, including observing a procedure or having the procedure performed on them.

### Acceptability

#### Acceptance

Participants’ initial reactions were overwhelmingly positive. HCWs, pregnant women, and their family members stated that introduction of the tool to health facilities should be done as soon as possible, that they would recommend it to others, and that the introduction would encourage more women to come to the clinic earlier in their pregnancies. The expected high acceptance of TraCer in the community was predicted to result in increased ANC uptake and attendance throughout pregnancy.

I think it’s good if you introduce it. It will help us to get the exact dates. Especially the mothers we are dealing with in the community, some of them will tell you that… “I can’t tell when my last periods were. I just realized that am pregnant.”

ANC clinic nurse IDI, Mariakani

First, if the tool is brought, it will give the women motivation to come to the clinic. They will be desiring to come to the clinic…

They will feel happy…because that tool is there. “You mean if I go to the clinic, I can see how my baby is doing?”

It [TraCer] will make you come. You won’t be saying let me wait for 2 months…

You will come early

Discussion in the pregnant women FGD, Rabai

Pregnant women felt that seeing the baby would give them an indication of the baby’s well-being and reduce their anxiety. The value of obtaining the GA would help them know the estimated date of delivery with more certainty. Other favorable features were the device’s safety to both mother and baby, and the short duration of the assessment. In particular, participants from rural areas suggested that TraCer would reduce the need to travel to the urban referral site to access ultrasound services, resulting in savings in both time and money.

Pregnant women and their families expressed that they would trust the results provided by TraCer. The ability for one to see the image “for themselves” during use of TraCer was emphasized as a major contributor to trust. This was contrasted to other clinical procedures such as listening to the fetal heartbeat using a fetoscope, which is assessed only by the health provider. A printout of the image after the procedure was suggested. Trust in the findings of TraCer also reflected pre-existing trust in HCWs in general. Participants assumed the device would already have been tested prior to introduction to ensure its efficacy and safety.

…Detecting the baby’s heartbeat…It means that the baby is alive…It means that he is doing well. So…it brings that confidence to the mother.

Clinical Officer IDI, Rabai

In my view, it will have reduced costs. Instead of traveling from here to Mariakani to queue there for a scan, and maybe I don’t have the ability to go there because of my pocket [ability to pay].

Male partners FGD, Rabai

Because I will be watching from the tablet when am being examined…because you are seeing, definitely you will trust the results.

Mothers and Mothers-in-law FGD, Mariakani

HCWs liked that TraCer would make it easier to obtain the GA, particularly for women with uncertain or unknown LMP. The ability to use new technology and give accurate GA estimation would boost their professional self-image and confidence in their services. The automated estimation of GA was viewed favorably, as it was perceived to be less susceptible to human error or interference by users. By providing simple GA assessment for all mothers in ANC clinics, TraCer would reduce workload at the few ultrasound facilities available because women requiring GA assessment only would not need a referral.

Overall, HCWs were optimistic that TraCer would find acceptance among pregnant women and community members. It would increase patient confidence in health providers and in their services, and would save them time and money spent traveling to referral facilities for ultrasound, ultimately leading to better retention of patients and continuity of care. HCWs expressed that many patients preferred health facilities that used advanced technologies, equating this to quality care.

…when we just want know the gestation age and fetal vitals it becomes more convenient than the ultrasound.

Doctor IDI, Mariakani

Most people like to work in a place where you feel good. You feel good working because there are machines, there are less challenges. …At least when handling a machine, like for me who I’ve not used an ultrasound… Aaah, I feel motivated. I feel I’ve arrived…

Clinical Officer IDI, Rabai

…we only have one ultrasound machine and only that one covering the whole hospital. Maternity cases, the wards, emergency department, outpatient clinics…and all patients to line up. And we only have only one sonographer doing all that. So it’s kind of overwhelming to the sonographer when you line up maternity patients only to know the gestational age.

Other HCW IDI, Mariakani

#### Hesitancy and Refusal

Although perceptions of TraCer were largely positive, some participants expressed preconditions that would need to be met before acceptance. The most frequent was the need to test the device’s accuracy, often through comparison with other GA assessment methods. Among HCWs, this meant a comparison with formal ultrasound, while community members suggested comparing the estimated date of delivery obtained from TraCer with the actual delivery date. Concerns about the performance and accuracy of TraCer were raised more often by urban than rural participants. Among HCWs, these concerns were more common among the higher skilled providers (ie, doctors and sonographers). HCWs also expressed that the procedure would have to be brief to avoid delays, which would result in rejection of its use.

Despite the general perception that health technology was viewed favorably, some participants felt such a new innovation would not easily be trusted, and that observing how TraCer performs over time before fully trusting results was necessary. Others suggested that fear and uncertainty regarding safety for the fetus could result in opting against use. Consequently, it was suggested that a principle of informed choice be practiced to ensure pregnant women were able to choose whether or not to be examined using the device.

Cause it’s something new, yeah. You can’t trust something new without actually trying it out. You have to put it to the test.

Doctor IDI, Mariakani

I’ll take the gestation age using the ultrasound machine then we just compare. We will compare. Because for now I just don’t know how accurate it [TraCer] is.

Other HCW IDI, Mariakani

…I will compare because, you know, the TraCer machine you have said it measures the Cerebellum. … And in our case, in the ultrasound we don’t use that. ...I have never used it. … I don’t know how accurate it is. But, maybe with time.

Other HCW IDI, Mariakani

You know, we Kenyans, when something new is introduced, we wait till the product has worked for a while. That’s when we start appreciating it

Male partners FGD, Rabai

### Feasibility

#### Proposed Approach to Clinical Implementation

HCWs, pregnant women, and their families described ANC clinics as the ideal location for clinical implementation, as it is the typical first point of contact with most pregnant women at the health facility. This would also contribute to consolidation of services for pregnant women at a single location, reducing movement of pregnant mothers within the facility and saving time. Some suggested that TraCer be placed permanently at the ANC clinic to ensure it is always available to the women.

Participants unanimously agreed that TraCer should be provided by the ANC nurse as the nurse is already the main provider of ANC and is believed to have the relevant background knowledge of reproductive anatomy. In addition, some doctors and clinical officers felt that they too should be familiar with the tool since they also assessed pregnant women with complications in outpatient and maternity units.

…when it is introduced, it has to be based at the ANC [antenatal care clinic] because all our new mothers coming for antenatal, that is the first place they come.

Other HCW IDI, Mariakani

I think all people who will be interacting with the pregnant mothers should be in position to use the tool effectively ‘cause we interact with the pregnant mothers at different levels and I think when we are able to attend to them, any place, anytime, that would be better off than just to be basing it at the maternal child health care clinic only.

Clinical Officer IDI, Mariakani

The need for training was emphasized by both community members and HCWs. All HCWs, regardless of prior training or education, felt confident that they would be able to use TraCer after appropriate training. There were contrasting views on the nature and intensity of the training needed. Most HCWs felt that because they had basic knowledge of anatomy and physiology, on-job training of a short duration focusing on how to use and how to interpret the results would suffice. However, two doctors suggested that more intensive training was necessary to address potential difficulties in locating the baby’s head in unusual fetal positions or in cases of multiple pregnancy. An additional recommendation was that other HCWs at the facility should be informed about the tool and its purpose to allow accurate communication to patients and community members.

…the nurse already [has] detailed [knowledge of the] anatomy of the woman’s abdomen and also when it comes to the uterus. So I think with that background information they have in their profession practice, I think it won’t be too difficult for it to require a very intensive training...

Other HCW IDI, Rabai

#### Concerns Regarding Implementation

Sustainable and consistent implementation were considered to be important by HCWs. HCWs raised concerns about a reliable supply of consumables needed and equipment maintenance. The device’s hardware was often viewed favorably as “portable,” “cordless,” “lightweight,” and “easy to carry around,” in contrast to conventional ultrasound machines. However, some HCWs highlighted the risks of theft or misplacing the device because of its small size and portability. If TraCer became unavailable when mothers came to expect it, they would be less inclined to return to the clinic. To achieve service consistency, there would need to be adequate staffing and clear policies and guidelines for its use.

Then those supplies, who is maintaining those supplies?…who is going to sustain? So who continues to supply this?…

Maternity nurse IDI, Mariakani

…the facility that is using the device, can they be able to maintain the device? In case of any breakdown…and are we going to have some trained personnel service the device in case it becomes faulty?…that’s what I was referring to as sustainability.

ANC clinic nurse IDI, Rabai

Misconceptions about TraCer were prevalent, especially among pregnant women and their families, but were also mentioned in 8 out of 18 interviews with HCWs. Many cited expectations of the device that were beyond its scope: the most frequent was that TraCer would provide detailed information about the well-being of the fetus and the mother. Other misconceptions included that the device would reveal fetal sex, confirm paternity, predict the exact date of delivery, that it could be used in the management of complications during labor and delivery, or that it could replace a formal obstetric ultrasound.

With that device, if a woman is pregnant, you will know everything that is happening insider her womb.

Male partners FGD, Mariakani

But with this one, I think there will be no need for the ultrasound because everything we’ll see it at the antenatal clinic.

ANC clinic nurse IDI, Rabai

Some participants understood the role of TraCer as primarily to provide GA estimation but expressed their desire for the tool to give more information.

…it will be good also if more research is done so that we know the state of the mother and the baby, how old her pregnancy is and how to take care of herself so that she has a safe delivery. This tool also to be guiding this mother on how to take care of herself it will be much better. It will be good.

Male partners FGD, Mariakani

## Discussion

### Summary of Findings

In this study, we assessed a low-cost, portable, and artificial intelligence (AI)-enabled ultrasound device in two Kenyan settings. Overall, the device concept was found to be highly acceptable to women, their families, and HCWs, and implementation at health care facilities was felt to be feasible. The introduction of the device was predicted to contribute to reduced anxiety around the stage of pregnancy and fetal well-being, increase ANC clinic attendance, increase confidence by women in HCWs, and potential time- and cost-savings. It was also predicted to boost HCWs’ professional self-image. The preferred users of TraCer were ANC nurses. There was a clear message that effective implementation requires adequate consideration on training, sustainability, and consistency of service. In addition, addressing potential misconceptions is a clear outcome of this work.

Various features of TraCer contributed to its acceptance. In particular, the value of seeing an image of the baby and the ability to see fetal heart activity were appreciated and strongly linked to trust of the device’s findings/results. The added value of “seeing” has been highlighted in other studies [23]. Device features such as the small size, portability, and absence of connecting cords were cited as strengths.

Previous studies conducted in sub-Saharan Africa highlight the positive attitude toward new technological innovations in health [24-26]. Despite little previous experience, HCWs showed high levels of acceptance toward new technologies owing to expectations that new tools would be easy to use, make work easier, and take less time [25,26]. This is also true for studies evaluating ultrasound devices [23,27,28]. Our study is unique in that it assessed a tool using AI to automate analysis, and that the tool is intended for a narrow use-case of GA assessment. Despite these features, the positive attitude toward the tool and the willingness to have it introduced was shared. The use of the specific device was viewed favorably and equated with quality, with HCWs expressing eagerness to learn its use. In our study, HCWs expressed confidence in automation and felt that the results would be more reliable due to reduction of human error and absence of interference from providers. This is an important finding relevant to future technologies incorporating AI in medical devices in these settings.

Some hesitancy, due to novelty, was also expressed, indicating the need for robust testing of performance and the effect of implementation over time. This is an important finding for implementation, demonstrating that immediate acceptance of new tools upon introduction cannot be expected. It is likely that the experience during early phases of implementation may influence eventual acceptability of novel tools.

There is increasing use of low-cost portable ultrasound in low-resource settings [29]; however, the majority of studies have been conducted in large tertiary hospitals with only a few conducted in rural facilities. Our study demonstrated higher acceptance of TraCer in the rural site. It is possible that this is due to the lack of any ultrasound service in that setting (Rabai) and the challenges associated with referral to the urban site. This unequal distribution of ultrasound services is common in sub-Saharan Africa [13], and sites for future implementation of TraCer and similar technologies should be carefully selected with this in mind. In contrast, the higher hesitancy in the urban site (Mariakani) could be a reflection of the higher existing technical skills and better understanding of potential pitfalls. Thus, HCWs with higher skill levels and prior experience with pregnancy ultrasound had more questions regarding accuracy and recommended more intensive training. They should therefore be provided with robust technical information and given ample time for training and testing.

This study suggests that implementation of TraCer in this setting will be feasible, but several potential barriers to effective implementation should be considered. Previous studies have demonstrated the feasibility of the use of small portable (compact) ultrasound devices, particularly for obstetric applications [30]. Similar to other studies [15], HCWs highlighted the need for equipment maintenance to ensure sustainability and consistency of the service. Service interruptions may be disappointing to staff and pregnant women and may affect their attitudes toward the tool [25].

HCWs were confident that they would be able to perform the procedure if given adequate training, but their views on the desired nature and duration of the training were varied. Other studies have evaluated combinations of didactics courses, hands-on instruction, supervised scanning, and lectures [31,32]. The duration of training for obstetric point-of-care studies in one review [31] ranged from 3 days to 3 months. Findings from our study are not sufficient to inform the design of a training package for TraCer, which will be evaluated during implementation. Addition of a training module on basic troubleshooting and repair of the device may be beneficial.

Like in other settings, misconceptions around the use of the technological innovations was common [28], with frequent reports of overestimation of its diagnostic potential [28,33]. It is possible that part of the positive attitudes toward TraCer reported in this study may have been influenced by these misconceptions and overestimation of the tool’s scope and function. Providing false reassurance of fetal and maternal well-being could contribute to delays in care-seeking for women, or may lead to blame in case of an adverse pregnancy outcome. Utmost care should be exercised to avoid HCWs becoming less thorough during clinical assessments or changing referral thresholds for further care due to such false reassurance of fetal well-being [27,33]. Thus, the role and scope of TraCer should be emphasized for both pregnant women and HCWs. Referral pathways for formal ultrasound assessment should remain open and indications for this made clear.

Nevertheless, the presence of a visible fetal heart beat is an important measure of fetal well-being. HCWs perceived that using TraCer would make assessment of the fetal heartbeat faster and easier to operate than a Pinard fetoscope or Doppler fetal monitor. We suggest that messaging around fetal status should acknowledge the confirmation that the baby is alive at the time of the examination, but clarify that this does not give reassurance of well-being.

### Proposed Structure of Implementation of TraCer

In view of the above findings, we propose that a fully validated and AI-driven TraCer device be used by nurses at the ANC clinic for assessment of all pregnant women. In this setting, TraCer would assess fetal viability and provide an accurate estimate of GA. Where there are indications for full obstetric ultrasound or other concerns for fetal well-being, women should be referred and encouraged to proceed for formal ultrasound assessment in line with current practice. With this approach, more women would have an accurate GA to inform subsequent decision-making and scheduling of effective ANC. Automated GA assessment using TraCer could reduce referrals for GA assessment only, giving higher-skilled ultrasound providers more time to focus on women needing specialist assessments. Such an approach has been suggested by other studies [13,34,35].

### Strengths and Limitations

Our study has a number of strengths. Data were collected from various participant groups and evaluated in the setting intended for use (ie, low-resource settings in LMICs). To increase the generalizability of findings, data were obtained from two health care facilities with differences in location (periurban vs rural), level (primary vs secondary), and availability of ultrasound services. The research team comprised skilled qualitative researchers and experts in maternal health and pregnancy ultrasound. The data collection and analysis team included researchers familiar with the local language and context.

The use of video role-play has been successful in eliciting rich data on acceptability and feasibility of the tool prior to clinical implementation. This approach has allowed an early evaluation, ensuring that the initial views of users are incorporated into further tool development and planning for actual clinical implementation. The study has also helped to understand baseline user expectations that can be used to measure clinical implementation indicators.

Although useful, video role-play also has limitations, as it may create an inaccurate perception of proposed clinical implementation. In this case, study participants felt that use of the device took very little time, an impression that could be created by an edited video. To overcome this potential limitation, further assessments during active clinical implementation of the tool are important.

Some of the research team had had interactions with study participants (HCWs) prior to data collection as part of preparations for a larger research study. This is important as it could have introduced some social desirability bias, with these responders expressing a more favorable view toward TraCer. This was mitigated by selecting the interviewer who had had the least prior interaction with the participant.

### Conclusion

We have shown that there is potential to implement AI-enabled ultrasound innovations in low-income settings, including a device that offers only a selected fetal assessment (in this case, GA). It is highly likely that the TraCer tool can be implemented in this and similar settings, and that users will find it valuable. Further device developments should ensure that the tool is simple and easy to use, and that results are obtained within a short time frame. Prior to clinical implementation, robust accuracy data must be provided and measures should be taken to ensure sustainability and consistency of the service. Clarity of messaging about the tool and its role in pregnancy is essential to prevent misconceptions and misuse. Our study points to further assessments required during the subsequent phases of clinical implementation, feasibility, acceptability, and device usability, as well as clinical validity and safety of the tool.

## Appendix

Multimedia Appendix 1 Codebook for data analysis.

## Abbreviations

AI: artificial intelligence
ANC: antenatal care
FGD: focus group discussion
GA: gestational age
HC: head circumference
HCW: health care worker
IDI: in-depth interview
LMIC: low- and middle-income country
LMP: last menstrual period
PRECISE: Pregnancy Care Integrating Translational Science, Everywhere
TCD: transcerebellar diameter
