# Supplementary material for: Acceptability and Feasibility of a Low-Cost Device for Gestational Age Assessment in a Low-Resource Setting: Qualitative Study
Source: JMIR Hum Factors. 2022 Dec 27;9(4):e34823. doi: 10.2196/34823 (PMC9832351; doi:10.2196/34823)
Supplement: Multimedia Appendix 1 [file humanfactors_v9i4e34823_app1.docx]

TraCer Kenya

Nodes

| Name | Description |
| --- | --- |
| **1. Importance of knowing gestational age** | Discussions of the importance of gestational age; why it is important to know an accurate gestational age |
| **2. Methods of gestational age estimation** | include mention of weaknesses of different methods (SFH, LMP), methods other than ultrasound also includes discussions of EDD |
| **3. Methods of pregnancy identification, signs of pregnancy** | How/when woman/health care worker knew the woman was pregnant Discussions of how women identify they are pregnant including any physical signs, at what point in gestation. For example, nurse did palpation at 22 weeks, LMP, hospital urine test |
| **4. Experience with other ultrasound methods & relevant technology** | Include responses to experience with smart phones and tablets, assessing “technological literacy” Interviewer: Smart phone? Respondent: Yes, I do. Interviewer: Have you ever observed an ultra sound (smile) Respondent: Those days I used to work in the maternity unit yes I used to. |
| **5. Perceptions of other ultrasound methods** | Perceptions of other ultrasound methods, may include discussions of cost of traditional ultrasound |
| **6. Ability for TraCer to be implemented into the system** | What will make it easier or harder to implement TraCer into the existing system may include discussions of preparation, training Getting ready to setup/start TraCer Applies most to health care managers and administrators, and some health care workers |
| Barriers | Factors that hindered use, such as concerns about workload, fears, safety, complexity, cost |
| Faciltators | Factors that enable use, such as workload, simplicity |
| Preferred location(s) for implementation | For example, at the hospital |
| Preferred provider(s) for implementation | For example, the nurse because she is the one examining us at the hospital |
| Training needs | Any mention on training for TraCer, who should do the training, how much training is needed etc. |
| **7. Perceptions of implementation of TraCer** | Perceptions of the device and its use |
| Acceptance of TraCer | Mentions of liking the device, happy about it, thinking that it’s good, feel that it would be helpful for the care of pregnant women Discussions about level of acceptance expected by patients/communities/other health care workers |
| Potential benefits of TraCer | Benefits we could expect after implementing TraCer into the system relationship with other providers, job prestige |
| Potential concerns related to TraCer implementation | Concerns once TraCer is implemented into the system this may include concerns around workload, |
| Recommend TraCer to others | Discussion of whether TraCer would be recommended to others or not |
| Refusals, rejection of TraCer | Refusals can be direct (woman declining it herself) or indirect (talking about other women declining it or it being optional) |
| Trust or mistrust of TraCer and its results | For example, the tool is said to be accurate. TraCer is not guessing. |
| **8. Features** | discussions of features/elements that were attractive (hardware) - ie seeing the fetal heart, showing women an image in real-time, complexity |
| **9. Misunderstandings of TraCer** | Discussions demonstrating misunderstandings of the TraCer device - ie. can determine sex, see baby’s wellbeing and fetal development, solve all complications, will tell the exact delivery date |
| **Other remarks** | Anything that does not fit clearly in another node |
